# The Sticky Resting Box, a new tool for studying resting behaviour of Afrotropical malaria vectors

**DOI:** 10.1186/1756-3305-7-247

**Published:** 2014-05-29

**Authors:** Marco Pombi, Wamdaogo M Guelbeogo, Katharina Kreppel, Maria Calzetta, Alphonse Traoré, Antoine Sanou, Hilary Ranson, Heather M Ferguson, N’Fale Sagnon, Alessandra della Torre

**Affiliations:** 1 Dipartimento di Sanità Pubblica e Malattie Infettive, Università di Roma “Sapienza”, Rome, Italy; 2 Institut Pasteur Fondazione Cenci Bolognetti, Rome, Italy; 3 Centre National de Recherche et Formation sur le Paludisme, Ouagadougou, Burkina Faso; 4 Institute of Biodiversity, Animal Health and Comparative Medicine, University of Glasgow, Glasgow, UK; 5 Vector Group, Liverpool School of Tropical Medicine, Liverpool, UK

**Keywords:** *Anopheles gambiae*, Malaria, Resting behaviour, Mosquito sampling, Sticky trap, Ecology

## Abstract

**Background:**

Monitoring densities of adult mosquito populations is a major challenge in efforts to evaluate the epidemiology of mosquito-borne diseases, and their response to vector control interventions. In the case of malaria, collection of outdoor-resting Anophelines is rarely incorporated into surveillance and control, partially due to the lack of standardized collection tools. Such an approach, however, is increasingly important to investigate possible changes in mosquito behaviour in response to the scale up of Insecticide Treated Nets and Indoor Residual Spraying. In this study we evaluated the Sticky Resting Box (SRB) - i.e. a sticky variant of previously investigated mosquito Resting Box, which allows passive collection of mosquitoes entering the box – and compared its performance against traditional methods for indoor and outdoor resting mosquito sampling.

**Methods:**

Daily collections were carried out in two neighbouring villages of Burkina Faso during rainy season 2011 and dry season 2012 by SRB located indoors and outdoors, and by Back-Pack aspiration inside houses (BP) and in *ad hoc* built outdoor pit-shelters (PIT).

**Results:**

Overall, almost 20,000 Culicidae specimens belonging to 16 species were collected and morphologically identified. Malaria vectors included *Anopheles coluzzii* (53%), *An. arabiensis* (12%), *An. gambiae* s.s. (2.0%) and *An. funestus* (4.5%). The diversity of species collected in the two villages was similar for SRB and PIT collections outdoors, and significantly higher for SRB than for BP indoors. The population dynamics of *An. gambiae* s.l. mosquitoes, as obtained by SRB-collections was significantly correlated with those obtained by the traditional methods. The predicted mean estimates of *An. gambiae* s.l. specimens/sampling-unit/night-of-collections was 6- and 5-times lower for SRB than for BP indoors and PIT outdoors, respectively.

**Conclusions:**

Overall, the daily performance of SRB in terms of number of malaria vectors/trap was lower than that of traditionally used approaches for in- and outdoor collections. However, unlike these methods, SRB could be set up to collect mosquitoes passively over at least a week. This makes SRB a promising tool for passively monitoring anopheline resting populations, with data presented here providing guidance for how to set up SRB-based collections to acquire information comparable to those obtained with other methods.

## Background

Monitoring densities of adult mosquito populations is a major challenge in efforts to monitor the epidemiology of mosquito-borne diseases, and evaluate the short and long term impacts of vector control interventions. In the case of malaria, most vector surveillance activities focus on sampling host-seeking and indoor-resting mosquitoes, with collection of vectors that are resting outdoors between feeding cycles being much less routine. However, characterization of the resting vector population, and in particular their endo/exophilic behavior, is crucial for evaluation of the likely success of malaria control measures and vector response to them. For example, the effectiveness of Indoor Residual Spraying (IRS) is critically dependent on the degree of endophily within target vector populations [1]. Furthermore, there is a need to assess whether the scale-up of IRS and use of Insecticide Impregnated Nets (ITNs) in recent years, [2] has generated selective pressures on vectors to become less endophilic. In fact, the use of such control measures has already been associated with a shift in vector species composition from endophilic, anthropophagic vectors towards more exophilic, zoophagic sibling species in some settings [3-7]. Whether such shifts in resting behaviour are also occurring within vector species is not yet known, but should it happen, could pose a problem for future control efforts where residual transmission is likely to largely occur in the outside environment.

However, methods to sample the resting fraction of Anopheline populations are far from optimal. Traditionally, indoor resting mosquitoes have been sampled by Pyrethrum Spray Catches (PSC) and active aspirations (e.g. by mouth and electric aspirators), and outdoor resting mosquitoes by *ad hoc* built Muirhead-Thomson pit-shelters [8]. Although they can be efficient, PSC present the limitations of causing significant disruption to householders, of being biased against pyrethroid resistant populations and of rendering the indoor environment unusable for further collections for several days. Active mouth aspiration collections require visual identification of vectors before capture, and thus are strongly affected by the skill of personnel involved. Back-pack aspirations provide a more standardized means of collection, but are still subject to variation due to the skill of the user and biased by residual insecticides in IRS-treated houses. Outdoor sampling inside pit-shelters is difficult to standardize and is complicated by the time required to build and maintain them particularly during the rainy season and by the risks associated to the possible presence of dangerous animals such as snakes. Thus, only a small number of pit-shelters, fixed in a few locations, are usually exploited for sampling, which may have limited ability to represent outdoor resting populations over wider geographic areas.

In recent years, alternative approaches based on the collection (via aspiration) of mosquitoes resting in small, portable containers distributed in the outdoor environment (e.g. resting boxes and clay-pots) have been tested with the aim of providing a more standardized trapping tool [9,10]. An advantage of these approaches is that the portable nature of the resting traps implies that they could be deployed in high numbers and in a wide variety of habitats, with minimal disruption to local residents. Although, clay pots have been shown to have good performances when tested against PSC indoors and pit-shelters outdoors in western Kenya [11], in general these approaches yielded substantially fewer mosquitoes than host-seeking collection methods [9,11-14].

In this study, we present a novel sticky variant of the mosquito Resting Box [9] (i.e. the Sticky Resting Box, SRB) designed to be more efficient, based on the assumption that lining the internal wall of the box with adhesive material will capture every mosquito that rests within the box, not just the fraction present at the time of collection by aspiration. Additionally, these traps could be less labor-intensive by allowing passive collection of mosquitoes entering the box over several days. We tested the capacity of the SRB to collect resting Anophelines and compared its trapping efficiency with that of traditionally used approaches for indoor and outdoor collections (i.e. Back-Pack aspirations inside houses and pit-shelters collections outdoors, respectively) in order to assess whether the SRB can provide a practical alternative tool for studying the resting behaviour of malaria vectors and other mosquito species.

## Methods

### Trap description

**Figure 1:**
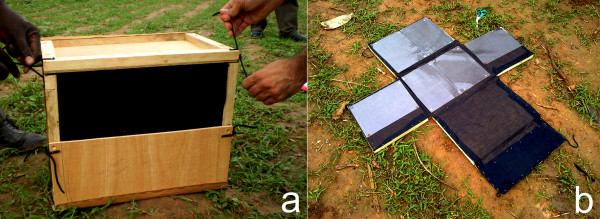
**The Sticky Resting Box (SRB). a)** Mounted trap showing the front entrance and the assembling system; **b)** Open trap during servicing, showing the position of sticky sheets in the inner walls of the trap.

An easy-to-package and to transport, demountable wooden Sticky Resting Box trap (45×33×35 cm, hereafter SRB) was designed (Figure 1a), based on the shape and size of Resting Boxes previously applied to collect resting *Anopheles* in Africa [9]. The SRB has a 45×15 cm opening at the upper front side and internal walls covered by black cotton cloth. Inside the trap, the lateral sides, with the exception of the frontal one, are lined with A4-acetate sheets manually coated with rat-glue (Figure 1b). A plastic container (15 cm diameter) filled with 1/2 liter of water was located inside the trap to assure high internal relative humidity.

### Field sampling procedure and mosquito processing

A comparative analysis of SRB performance against traditional approaches to collect mosquitoes indoors (i.e. aspirations by CDC backpack model 1412, BioQuip Products, Inc., hereafter BP) and outdoors (i.e. manual aspirations in Muirhead-Thomson pit-shelters, hereafter PIT) was carried out in two villages (Koubri, 12°12’N - 1°22’W, and Goden, 12°25’ N – 1°21’W) in the Ouagadougou area of Burkina Faso in West Africa. Collections were carried out twice weekly in 4 family compounds in each village from the early rainy season to the early dry season (July-December 2011, hereafter RS-2011), and during the dry season only in Goden (April –June, hereafter DS-2012).

In each compound, collections were carried out on the same day during early afternoon (i.e. approximately 2:00–4:00 PM) by: i) 5-minute long aspiration/house by BP performed by the same person in two inhabited single-room houses in each compound; ii) 1 SRB/house located on the ground (and oriented in a way that the entrance was partially shaded in order to simulate a hidden resting site) in the same two houses where BP collections were conducted; iii) aspiration in 1 *ad*-*hoc* built PIT located at a distance of about 50 m from each sampled compound; and iv) SRBs (2 SRB in RS-2011, and 8 in DS-2012, in order to take into account the very low mosquito densities expected during dry season) located at 5–10 m from the closest house and from each other and placed with the entrance partially hidden, as done for indoor sampling. Collections made by either 1 BP, 1 PIT, or 2 SRBs took approximately the same amount of time (5–7 min).

Mosquitoes glued on sticky sheets were removed by cutting out a small sheet fragment around them and washing this with acetone for 2 minutes. All collected mosquitoes were morphologically identified under a stereomicroscope [15] and separated by species, sex and gonotrophic stage and stored in individual tubes containing silica-gel for molecular identification. *Anopheles gambiae* s.l. mosquitoes were subdivided into two equal groups and molecularly identified by PCR-RFLP [16] in CNRFP and by PCR-SINE X [17] in “Sapienza” University.

The experimental activities here described have been performed with the approval of Burkina Faso ethics committee “Comité d’ethique pour la recherché en santé”, in agreement with Ministry of Health and Ministry of Research (approval n. 2011-6-34, issued June, 6^th^ 2011).

### Evaluation of sticky sheet performance

All SRB-collections were carried out using manually coated sticky-sheets. However, we compared the performance of these with commercially available adhesive cardboard panels for the collection of insects caught by UV-traps (GEA Italy) and did not find any significant difference for *An. coluzzii* mosquitoes (data not shown). Therefore, the experiment below was carried out using the commercially available panels.

Although field collections were carried out by replacing sticky sheets daily in order to get results directly comparable with those obtained by daily BP and PIT aspirations, the SRB was conceived to provide the opportunity of continuous passive data collection over multiple days without the need for servicing. To evaluate this feature, we assessed the adhesiveness of the panels over time and tested the DNA quality of the specimens that had been glued to them for several days. To do this, SRBs were set up in each of three 3.4 m^3^ outdoor cages in CNRFP’s courtyard. Four SRBs were placed in each cage, and left for 7 days. On days 1, 3, 5 and 7, 100 blood fed laboratory reared *An. coluzzii* females were released into the outdoor cages and the number that became trapped in SRB recorded as a means of testing whether adhesiveness reduced over time.

Additionally, the DNA quality of the specimens that had been glued for different lengths of time was assessed by manually sticking 319 laboratory-reared *An. coluzzii* mosquitoes onto 16 adhesive panels in 4 SRBs, which were then kept outdoors for 1, 3, 5, and 7 days. At each time-point, glued mosquitoes were removed from 1 panel/SRB, freed from glue by acetone, and stored in silica-gel for >60 days (in order to replicate routine sample manipulation during field experiments) and identified by PCR protocol [17].

### Statistical analysis

#### i. Mosquito abundance

Variation in mosquito abundance among collection methods was investigated using Generalized Linear Mixed Models [18]. Separate analyses were conducted for each village and season, respectively.

In these analyses, ‘collection method’ was fitted as a main effect, with compound, trapping week and sampling unit ID fitted as random effects. Mosquito abundance data are typically overdispersed, consequently a Poisson or Negative Binomial distribution was used to model the data depending on their fit to these distributions (with fit being assessed visually through inspection of fitted residuals).

The predicted mean numbers of Anophelinae and Culicinae mosquitoes collected by each trapping method were obtained by extracting and exponentiating the coefficients and associated standard errors predicted from statistical models.

Evaluation of the consistency of the relationship between SRB catches and PIT and BP aspirations was carried out by calculating the Spearman’s Correlation Coefficient between their mean daily captures (as predicted from GLMMs).

#### ii. Mosquito diversity

The Gini-Simpson’s Diversity Index 1-D was applied to evaluate species diversity, using the formula:

$$1-D=1-\frac{\sum n_{i}\left(n_{i}-1\right)}{N\left(N-1\right)}$$

with the 95% confidence limits of this index were calculated as: $\pm 2\sqrt{\frac{\sum {\left(\frac{n_{i}}{N}\right)}^{2}-{\left(\sum {\left(\frac{n_{i}}{N}\right)}^{2}\right)}^{2}\;}{N\left(N-1\right)}}$ where *n*_*i*_ is the abundance of the species *i* and *N* is the total number of individuals per sample [19]. The Simpson’s index of evenness (*E*) was calculated to obtain a measure of the relative abundance of the different species in the sample, using the formula:

$$E=\frac{1-D}{1-D_{\max}}$$

where *D*_*max*_ is the highest value of the 1-D index for the given number of species and the sample size [19].

#### iii. Mosquito vector species composition

Analysis of variation in the species composition of malaria vectors (i.e. *An. gambiae* complex species) were calculated on the basis of samples pooled per trap type over a one week period (2 days/week).

To test for differences among collection methods in the species composition of *An. gambiae* s.l. sampled, the proportion of the subsample that was identified as belonging to *An. coluzzii* (via PCR) was calculated. Generalized Linear Mixed Models were used to test the association between the proportion of *An. coluzzii* in the sample, with collection method fitted as a fixed effect, and sampling week as a random effect.

All statistical analyses were conducted in R, v2.15.3 [20] using the lme4 package [21] for generalized linear mixed effects models.

## Results

**Figure 2:**
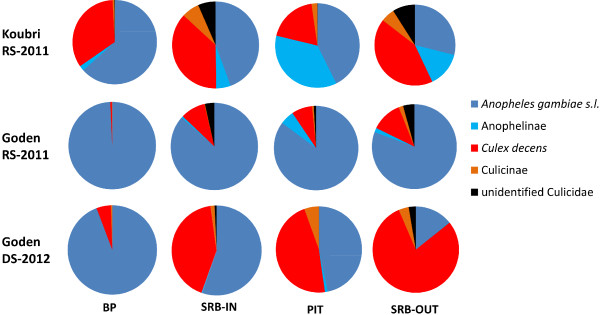
**Relative frequencies of mosquito genera/species collected by different trapping methods in two villages of Burkina Faso.** Samples from Koubri (N=6005) and Goden (N=7720 and N=5930 during year 2011 and 2012, respectively). SRB-IN=sticky resting box collections indoors; BP=back-pack aspirations indoors; SRB-OUT=sticky resting box collections outdoors; PIT=collections in outdoor pit-shelters; RS-2011=July-December 2011 sampling; DS-2012= April-June 2012 sampling.

Overall, 19,655 Culicidae specimens belonging to 16 species were collected during the study (12 species in SRB-IN; 11 in BP; 12 in SRB-OUT; 13 in PIT); 99.5% of these were successfully morphologically identified (Additional file 1). Figure 2 shows the relative frequencies of species collected in each village by BP or SRB indoors (hereafter SRB-IN) and by PIT or SRB outdoors (hereafter SRB-OUT). Anophelinae represented 74% of the total sample and included *An. gambiae* s.l. (67% of the total Culicidae), *An. funestus* (4.5%, mostly found in PITs during rainy season collections in Koubri), *An. rufipes* (2.3%), *An. muscinioi* (0.03%), *An. nili* (0.02%) and *An. pharoensis* (0.01%). Culicinae were represented mostly by *Culex decens* (23% of total sample), which was the most abundant mosquito species found in outdoor collections during the dry season in Goden (9.3% of the total). Each method collected Anophelinae females and males in comparable proportions (with few exceptions), while SRB consistently collected more Culicinae females than males during RS-2011 (Table 1).

**Table 1:** Daily predicted mean estimates (±SE) of Anophelinae and Culicinae mosquitoes collected by Sticky Resting Box and by traditional collection methods

**Village**	**Method**	**Gender**	**Anophelinae**			**Culicinae**		
			**Mean**	**+SE**	**-SE**	**Mean**	**+SE**	**-SE**
Koubri	BP	♀	2.85	4.09	1.99	0.92	1.49	0.57
RS-2011		♂	1.56	2.36	1.03	0.60	0.93	0.39
	SRB-IN	♀	0.82	1.18	0.57	0.93	1.48	0.59
		♂	0.35	0.54	0.23	0.15	0.24	0.09
	PIT	♀	10.49	14.89	7.39	1.55	2.20	1.09
		♂	8.20	11.57	5.81	0.77	1.47	0.47
	SRB-OUT	♀	1.72	2.37	1.25	1.25	1.76	0.89
		♂	0.93	1.28	0.67	0.27	0.51	0.14
Goden	BP	♀	4.46	7.22	2.75	0.06	0.09	0.04
RS-2011		♂	2.55	4.11	1.58	0.01	0.02	0
	SRB-IN	♀	0.88	1.44	0.54	0.14	0.19	0.10
		♂	0.37	0.60	0.23	0.02	0.04	0.01
	PIT	♀	9.00	13.7	5.91	0.68	1.02	0.45
		♂	13.32	18.34	9.67	0.65	1.21	0.35
	SRB-OUT	♀	2.02	3.05	1.34	0.58	0.83	0.41
		♂	1.56	2.16	1.13	0.03	0.06	0.01
Goden	BP	♀	5.58	10.00	3.11	0.23	0.35	0.15
DS-2012		♂	1.37	2.45	0.77	0.36	0.59	0.22
	SRB-IN	♀	0.45	0.81	0.25	0.24	0.37	0.15
		♂	0.12	0.22	0.06	0.41	0.68	0.25
	PIT	♀	0.28	0.55	0.14	0.20	0.22	0.10
		♂	0.09	0.27	0.03	0.1	0.24	0.04
	SRB-OUT	♀	0.12	0.23	0.06	0.27	0.54	0.14
		♂	0.01	0.03	0	0.63	1.41	0.28

*SRB*-*IN* Sticky resting box indoors, *SRB*-*OUT* Sticky resting box outdoors, *BP* Back Pack aspirators indoors, *PIT* Pit shelters outdoors, *RS-2011* July-December 2011 sampling, *DS-2012* April -June 2012 sampling.

♀= mosquito females, ♂= mosquito males.

The diversity of species collected was significantly higher for SRB-IN (Simpson’s index= 0.56±0.02) than for BP (0.20±0.04), while it did not vary between SRB-OUT (0.49±0.06) and PIT collections (0.53±0.03), nor between SRB collections indoors *versus* outdoors. The relative abundance of different species did not vary among SRB-IN (Evenness index=0.53), SRB-OUT (0.59) and PIT (0.57), but was relatively lower for BP (0.21).

**Figure 3:**
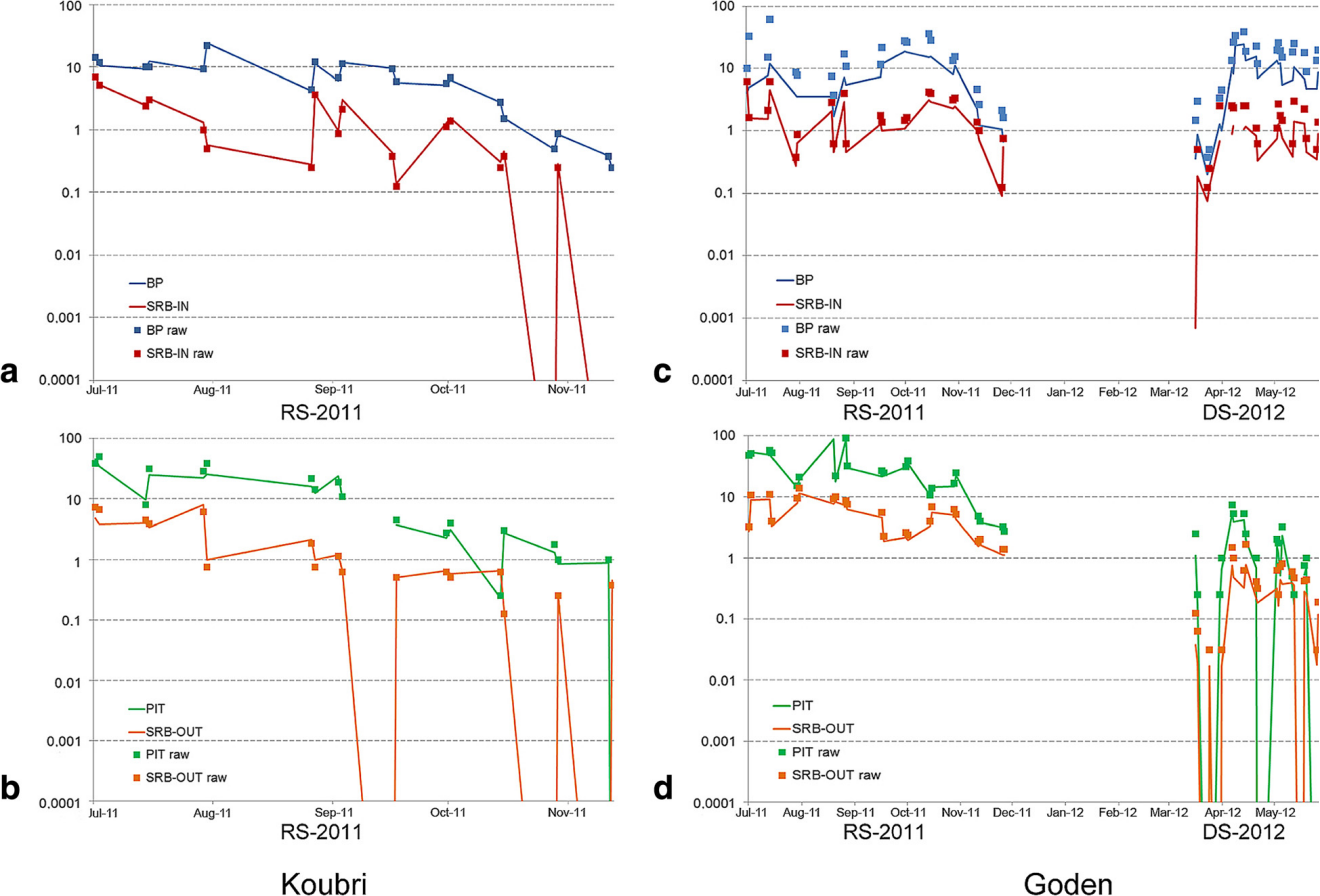
**Population dynamics of *Anopheles gambiae *s.l. in two villages of Burkina Faso during 2011 and 2012 samplings.** Blue and red solid lines correspond respectively to predicted mean estimates of daily catches of BP and SRB-IN collections in Koubri **(a)** and Goden **(c)**. Green and orange lines: PIT and SRB-OUT collections in Koubri **(b)** and Goden **(d)**. Solid squares represent daily mean catches based on raw data. SRB-IN=sticky resting box collections indoors; BP=back-pack aspirations indoors; SRB-OUT=sticky resting box collections outdoors; PIT=collections in outdoor pit-shelters; RS-2011=July-December 2011 sampling; DS-2012= April-June 2012 sampling.

The consistency of SRB performance with respect to traditional methods for trapping indoors (BP) and outdoors (PIT) was assessed by testing the strength of correlation between their daily predicted mean catches of *An. gambiae* s.l. mosquitoes. Strong positive correlations were found both between SRB-IN and BP collections and between SRB-OUT and PIT collections for villages and seasons, despite occasional fluctuations that were observed in few indoor and outdoor collections in Koubri during RS-2011, where SRB catches were much lower than BP ones (Figure 3; Table 2).

**Table 2:** **Spearman**’**s correlation coefficients of daily predicted mean estimates of *An. gambiae *** **s.l. mosquitoes caught by Sticky Resting Box and by traditional collection methods**

**Village**	**Season**	**Methods**	**N**	**Spearman’s ρ**	**2-tails p-value**
Koubri	RS-2011	BP vs. SRB-IN	20	0.818*	0
		PIT vs. SRB-OUT	19	0.772*	0
Goden	RS-2011	BP vs. SRB-IN	22	0.571*	0.006
		PIT vs. SRB-OUT	22	0.468*	0.028
	DS-2012	BP vs. SRB-IN	21	0.725*	0
		PIT vs. SRB-OUT	22	0.662*	0.001

* = significant correlation.

*SRB*-*IN* Sticky resting box indoors, *SRB*-*OUT* Sticky resting box outdoors, *BP* Back Pack aspirators indoors, *PIT* Pit shelters outdoors, *RS-2011* July-December 2011 sampling, *DS-2012* April -June 2012 sampling.

**Figure 4:**
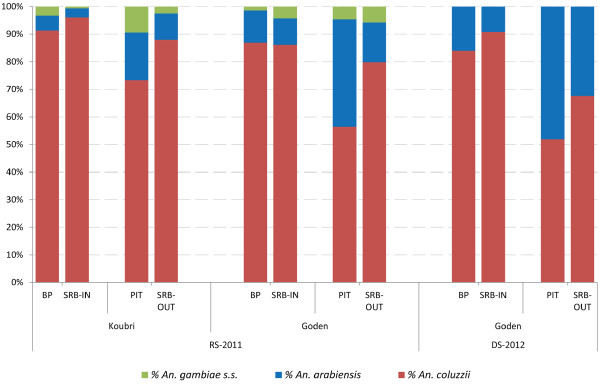
**Relative frequencies of members of the *Anopheles gambiae *complex collected by different trapping methods in two villages of Burkina Faso.** SRB-IN=sticky resting box collections indoors; BP=back-pack aspirations indoors; SRB-OUT=sticky resting box collections outdoors; PIT=collections in outdoor pit-shelters; RS-2011=July-December 2011 sampling; DS-2012= April-June 2012 sampling.

Among the 13,195 *An. gambiae* s.l. mosquitoes collected, 9,022 were processed for PCR-identification as follows: all specimens collected in Koubri (N=2,199), and 6,823 out of the 10,372 collected in Goden (all the 1,928 collected by SRB, and 2,809/5,508 and 2,086/2,884 from BP+PIT collections in 2011 and 2012, respectively). Of these 93% were successfully identified: *An. coluzzii*[22] (previously known as M molecular form, [23]) was the most abundant taxon (79%), followed by *An. arabiensis* (18%) and *An. gambiae* s.s. (previously known as S molecular form, 3%), which was only found during the rainy season (Figure 4); 14 *An. coluzzii* × *gambiae s.s*. hybrids (4 in Goden, of which 2 indoors and 2 outdoors and 10 in Koubri, 9 indoors and 1 outdoors) and 1 *An. coluzzii* × *arabiensis* hybrid were also found, corresponding to a relative frequency of 0.17% and 0.01%, respectively.

Focusing the analyses on the *An. gambiae* s.l. sample, which included the prevailing malaria vector species in the two villages, there was significant variation between collection methods in the abundance of mosquitoes caught (Table 3). Overall, SRB consistently collected fewer individuals, i.e. 5–12 times less than BP indoors, and 2–5 times less than PIT outdoors depending on gender and female gonotrophic stage.

**Table 3:** **Daily predicted mean estimates (±SE) of *An. gambiae *** **s.l. mosquitoes caught subdivided based on gender, female gonotrophic stage and sampling method**

**Position**	**Status**	**Method**	**Mean**	**+SE**	**-SE**	**Difference between methods**	**Ratio**
Indoor	All	BP	6.5	2.71	1.91	χ^2^= 31.12; p<0.001	6
		SRB-IN	1.08	0.45	0.32		
	Unfed	BP	1.42	0.56	0.4	χ^2^=28.81; p<0.001	5
		SRB-IN	0.31	0.13	0.09		
	Fed	BP	0.95	0.43	0.29	χ^2^=34.9; p<0.001	12
		SRB-IN	0.08	0.04	0.02		
	Gravid	BP	1.96	0.84	0.59	χ^2^=29.38; p<0.001	6
		SRB-IN	0.3	0.13	0.09		
	Male	BP	1.86	0.85	0.58	χ^2^=31.96; p<0.001	7
		SRB-IN	0.27	0.13	0.09		
Outdoor	All	PIT	3.37	2.17	1.32	χ^2^=24.16; p<0.001	5
		SRB-OUT	0.68	0.42	0.26		
	Unfed	PIT	0.57	0.34	0.21	χ^2^=15.93; p<0.001	2
		SRB-OUT	0.26	0.15	0.09		
	Fed	PIT	0.32	0.24	0.14	χ^2^=16.25; p<0.001	5
		SRB-OUT	0.07	0.05	0.03		
	Gravid	PIT	0.61	0.36	0.23	χ^2^=22.08; p<0.001	5
		SRB-OUT	0.13	0.07	0.05		
	Male	PIT	1.32	1.18	0.62	χ^2^=20.50; p<0.001	8
		SRB-OUT	0.16	0.14	0.07		

The SE and the differences (Chi-square and p-value) between trap types are calculated with respect to the predicted mean difference. Ratio is calculated dividing the predicted mean value of the first method per the second method.

*SRB*-*IN* Sticky resting box indoors, *SRB*-*OUT* Sticky resting box outdoors, *BP* Back Pack aspirators indoors, *PIT* Pit shelters outdoors.

**Figure 5:**
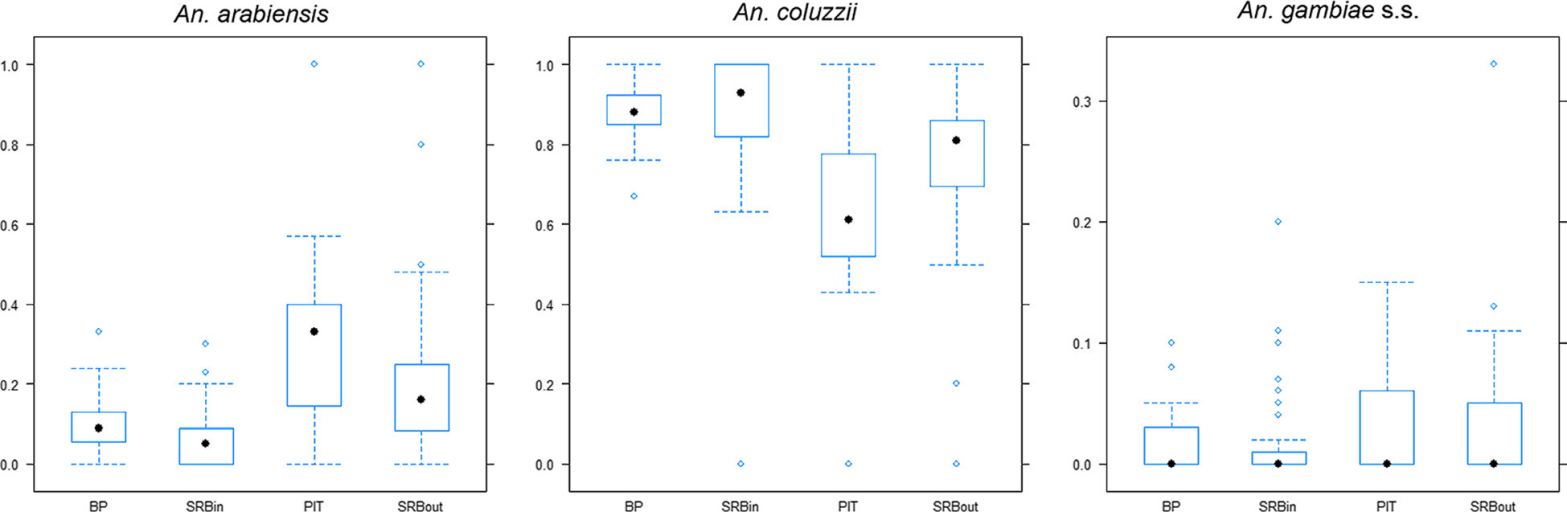
**Overall proportion of *An. gambiae *s.l. taxa per weekly sample per trapping method.** Solid dots represent the median rate for each trap type. Empty circles represent outliers. Bottom and top of the box show the 25^th^ and 75^th^ percentile respectively. Whiskers show maximum value or 1.5 times the interquartile range – whichever is the smaller. SRB-IN=sticky resting box collections indoors; BP=back-pack aspirations indoors; SRB-OUT=sticky resting box collections outdoors; PIT=collections in outdoor pit-shelters.

*Anopheles coluzzii* was proportionally more abundant in indoor collections (total mean proportion indoors and outdoors 0.87 and 0.68, respectively), while *An. arabiensis* (total mean proportion indoors and outdoors 0.09 and 0.26, respectively) and *An. gambiae s.s*. (total mean proportion indoors and outdoors 0.02 and 0.04, respectively) in outdoor ones (Figure 5). Differences in mean relative proportions of *An. gambiae* taxa are observed between PIT and SRB-OUT (*An. coluzzii*: Chi-square 22.0; p<0.001), but not between BP and SRB-IN, demonstrating that SRB collections are fully comparable to the indoor gold-standard, but not the outdoor one.

Strong differences are observed in the daily mean predicted numbers of *An. coluzzii*, *An. gambiae* and *An. arabiensis* females and males collected by each approach (Additional file 2), with higher values for BP and PIT compared to SRB for *An. arabiensis* and *An. coluzzii. Anopheles gambiae* s.s. catches were so low that no significant difference between trap performances could be established for this species. A high variability of mean numbers of mosquitoes collected with the same method in different villages and seasons is also observed, in particular for *An. arabiensis*.

Finally, the evaluation of the feasibility of weekly (instead of daily) servicing of SRB showed that the adhesiveness of the sticky panels after 7 days was not significantly lower than after 1, 3 or 5 days (Additional file 3). Moreover, specimens glued for 7 days on the panels yielded successful molecular PCR-identification in 86% of cases, with no significant differences with those glued for shorter periods.

## Discussion

The results of the longitudinal sampling study carried out to compare the performance of the newly designed Sticky Resting Box (SRB) show that this may represent a good tool for monitoring the resting fraction of Afrotropical mosquito populations and possible shifts in species-specific indoor/outdoor resting behavior.

First, SRB was shown to perform quite consistently with traditional collection methods with reference to: i) the relative overall densities of Anophelinae collected; ii) the relative proportions of Anophelinae females and males collected during the rainy season (while more females than males were found in SRB during the dry season); iii) *An. gambiae* species population dynamics. Moreover, SRB collected high genera/species diversity, comparable to that observed in PIT collections outdoors, but much higher than that attained by BP aspirations indoors, which appear to strongly underestimate the indoor presence of species other than those of the *An. gambiae* complex. These features make SRB potentially useful for faunistic and explorative purposes in areas where the presence of mosquito species and/or their phenology are unknown. Interestingly, for instance, a very high abundance of *Culex decens* (a container-breeding Afrotropical species, potentially implicated in the transmission of arboviruses, such as Rift Valley Fever, [24]) was revealed by all methods, with the relative abundance of the species being much higher in collections by SRB, particularly during the dry season.

Undeniably, daily collections by SRB were significantly less efficient in terms of malaria vectors collected per trap/night than traditional collection methods: indoors SBB collected 1/6 *An. gambiae* s.l. females (and 1/7 of males) captured by BP aspiration, while outdoors it collected 1/5 of the of females (and 1/8 of males) captured by PIT trap aspirations. The lower indoor efficiency was expected, as the relatively small SRB compete with several other alternative indoor resting places, which are scanned during the BP aspirations. Outdoors, SRB collected <2 individuals/day, similar to results obtained with other types of containers used to attract outdoor resting mosquitoes (e.g. resting boxes and clay-pots, [10-14]). However, SRB do not need daily aspiration of resting mosquitoes (which all other methods require), since the sticky panels maintain their adhesive properties for at least 7 days and the carcasses and the DNA of individuals glued in SRBs for up to 7 days is still suitable for morphological and molecular identifications. This implies that, with no increase in the economical and working effort needed, SRB can continuously collect mosquitoes during a whole week, thus becoming equally (or even more) efficient in terms of numbers of individuals collected per trap/night than daily aspirations inside dwellings or pits performed on a weekly basis. Due to the malaria vector species composition in the study area, the above calculation was only done for species of the *An. gambiae* complex. It is, however, important to note that few *An. funestus* individuals were also found in SRB (Additional file 1): while indoors their relative abundance in SRB was very low but comparable to that observed in BP collections, outdoors it was much lower in SRB than in PIT. In fact, PIT collected 95% of the overall *An. funestus* samples (most of which in Koubri), a result which merits further investigation, as previous data showed a high endophily of this species in the same area [25,26]. A possible explanation of the different performance of PIT with respect to SRB in collecting *An. funestus* could be also due to the potential effect of the glue, which could selectively attract (or repel) certain mosquito species affecting their propensity to enter in the box or rest on its surface. Further experiments are needed to evaluate the species-specific effect of the glue on mosquito resting behaviors.

Furthermore - as opposed to the traditional collection methods - SRB represents a “passive” collection approach, which is not restricted to the period of active aspirations and continuously collects mosquitoes overtime, thus providing estimates of relative densities of resting mosquitoes during an entire week. The use of glue instead of aspiration represents the great advantage of SRB compared to other devices used to collect outdoor resting mosquitoes, all of which need the active aspiration of individuals resting inside the devices [9,10]. In addition to these advantages, SRB also: i) is a standardized method not biased by the skill of the operator; ii) is cheap (estimated cost <20 €), can be easily built locally and can thus be deployed in high numbers; iii) can be equipped either with manually prepared glue-sheets (cheap and locally made), or with commercially adhesive panels (more expensive, but ready-to-use); iv) does not require power supply; v) is easy to dismount and transport; vi) is durable under field conditions); vii) is environmentally safe (although small animals, such as lizards and geckos, were occasionally found stuck in the SRB). Importantly, the glue on the sticky sheets was shown to be sufficiently strong to prevent trapped insects from being eaten by most common predators (e.g. ants or even lizards). It is, however, relevant to highlight that the recovery of mosquito specimens from the adhesive sheets is a more time-consuming procedure than the simple recovery of those collected by aspirators, which may complicate the sample scheme when the number of specimens is high. Moreover, while the DNA of glued samples has been proved to allow molecular identification, it is likely not optimal for high throughput genotyping and sequencing studies.

The results show that SRB could represent a valuable new tool for monitoring the exophilic fraction of anopheline populations and, possibly, their *Plasmodium* infections rates, thus addressing a recognized weakness in the surveillance of malaria vector populations, due to the problem of locating adults in their highly dispersed, natural resting sites [8]. In fact, building a large numbers of PITs in the vicinity of a village is environmentally disruptive and impractical in many situations, and alternative methods such as aspiration of vegetation or inside different types of containers are time-consuming and not very effective. Interestingly, the analysis of gonotrophic stages of *An. gambiae* s.l. females collected in SRB (Table 3) revealed that SRB may also represent a promising approach to study the feeding behavior (and its possible shifts) in the outdoor environment. As already mentioned, SRB could also be successfully used for monitoring the endophilic fraction of mosquito populations, with the additional advantage to collect a higher diversity of species compared to BP. Therefore, on the whole, SRB opens up the possibility to more easily investigate differences in the resting behavior of mosquito species, as well as possible shifts in relation to ecological changes and/or indoor insecticide treatments and use of insecticide-impregnated bed-nets. Notably, these aspects are a major challenge for the present and future planning and implementation of strategies aimed to reduce malaria transmission.

With reference to members of the *An. gambiae* complex, it is also interesting to note that the relative proportions of each species collected indoors by SRB are well correlated with those found in BP, implying that historical data obtained by BP collections can be compared to novel ones obtained by indoor-SRB, without any bias, at least in our study area. However, this is not true for outdoor sampling, where SRB collections yield proportionally less *An. coluzzii* than PIT. This may be due to a different intrinsic sampling capacity of the two outdoor approaches for the different species, or to actual differences in species composition at a different distance from the inhabited compounds (i.e. 50 m for PIT and <10 for SRB-OUT). Although it is not possible to know which proportion more closely reflects the actual species relative frequencies in the field, it is reasonable to assume that using the same sampling device (i.e. the SRB) would allow a more straightforward comparison of the endophilic/exophilic behavior of the species, than comparing results obtained by two different approaches, such as BP and PIT. In the study area, the results obtained show higher endophily in *An. coluzzii* than in *An. arabiensis* and *An. gambiae* s.s. Even though the low density of *An. gambiae* s.s. may affect the significance of these results, this may be the first evidence of a difference in resting behavior between *An. coluzzii* and *An. gambiae* s.s. On the other hand, the higher exophily of *An. arabiensis* is in agreement with other observations in the same area (Goundry village, Costantini C., personal communication), as well as with publications from other geographical regions, reporting the high plasticity of resting and feeding behavior of this opportunistic species [27-30]. This is not the case for *An. coluzzii* (i.e. M molecular form) that, even if in some circumstances is able to shift its feeding behavior exploiting bovine hosts in the absence of the preferred humans, seems to be consistently characterized by a high endophilic resting behavior [31].

It is interesting to note that collection sites lie within the area where the presence of an exophilic *An. gambiae* cryptic subgroup (i.e. GOUNDRY) has been hypothesized based on high frequencies of M/S heterozygous IGS-patterns in larval samples [32]. Despite the fact that we did not specifically genotype our samples for assessing the possible presence of GOUNDRY, the low frequency (0.17%) of *An. coluzzii*X*gambiae* adults (i.e. M/S heterozygotes) observed in the present study do not confirm the presence of this subgroup during the collection periods, despite the large number of specimens collected outdoors.

## Conclusion

The results presented demonstrate that SRB is an efficient new tool for passively monitoring *An. gambiae* s.l. species resting both indoors and outdoors in the study area and provide guidance for how to set up SRB-based collections to acquire information comparable to those obtained with traditional methods. However, as different resting devices have been shown to have varying collection efficiencies in different geographic areas [9,11-14], it is important to emphasize that calibration of SRB with other methods is recommended in ecological settings where it is planned to be used for the first time, in order to confirm the validity of SRB as a new reference standard for malaria vector monitoring.

## Abbreviations

SRB: Sticky Resting Box; SRB-IN: Sticky resting box placed indoors; SRB-OUT: Sticky resting box placed outdoors; BP: Back-pack aspirations indoors; PIT: Muirhead-Thomson pit-shelt; RS-2011: July-December 2011 sampling period; DS-201: April -June 2012 sampling period.

## Competing interests

The authors declare that they have no competing interests.

## Authors’ contributions

AdT, HMF, HR, MP, NFS and WMG conceived the study; MP and WMG participated in its design, coordination and field sampling; AS, AT and MC participated in molecular identification; KK and MP analyzed the results; AdT, HMF and MP drafted the manuscript. All authors contributed to and approved the final manuscript.

## Supplementary Material

Additional file 1 **Total number of mosquitoes collected in the villages of Goden and Koubri, Burkina Faso, separated per sex and species.** Taxa belonging to species complexes and groups are not detailed. Collections were carried out twice/week in 4 compounds/village, as follows for each compound: 1 aspiration/hut by back-pack aspirators (BP); 1 sticky resting box/house (SRB-IN); aspiration in 1 pit-shelter (PIT); 2 sticky resting boxes/compound outdoor (SRB-OUT) in rainy season 2011 (RS-2011) e 8 in dry season 2012 (DS-2012). N=number of sampling units/collection day. RS-2011=July-December 2011 sampling; DS-2012= April-June 2012 sampling. Click here for file

Additional file 2 **Daily predicted mean estimates (±SE) of individuals of species of the *An. gambiae *** **complex collected by Sticky Resting Box and traditional collection methods.** SRB-IN= Sticky resting box indoors; SRB-OUT= Sticky resting box outdoors; BP=Back Pack aspirators indoors; PIT=Pit shelters outdoors; RS-2011=July-December 2011 sampling; DS-2012= April-June 2012 sampling. ^§^ = non-significant difference between collection methods. Click here for file

Additional file 3 **Performance of SRB adhesive sheets over time.** Percentage of *An. coluzzii* females stuck on sticky resting boxes exposed to outdoor conditions for 1, 3, 5 and 7 days. Chi-square and p-values for pair comparisons are reported. Click here for file
