# Supplementary material for: The Sticky Resting Box, a new tool for studying resting behaviour of Afrotropical malaria vectors
Source: Parasit Vectors. 2014 May 29;7:247. doi: 10.1186/1756-3305-7-247 (PMC4049408; doi:10.1186/1756-3305-7-247)
Supplement: Additional file 1 — Total number of mosquitoes collected in the villages of Goden and Koubri, Burkina Faso, separated per sex and species. Taxa belonging to species complexes and groups are not detailed. Collections were carried out twice/week in 4 compounds/village, as follows for each compound: 1 aspiration/hut by back-pack aspirators (BP); 1 sticky resting box/house (SRB-IN); aspiration in 1 pit-shelter (PIT); 2 sticky resting boxes/compound outdoor (SRB-OUT) in rainy season 2011 (RS-2011) e 8 in dry season 2012 (DS-2012). N=number of sampling units/collection day. RS-2011=July-December 2011 sampling; DS-2012= April-June 2012 sampling. [file 1756-3305-7-247-S1.docx]

**Additional file 1**

| Sampling | Village | Year | Position | Method  (N) | Gender | Anophelinae | *Anopheles gambiae* s.l. | *Anopheles funestus* | *Anopheles rufipes* | *Anopheles muscinioi* | *Anopheles nili* s.l. | *Anopheles pharoensis* | *Culicinae* | *Culex decens* | *Culex nebulosus* | *Culex cinereus* | *Culex quinquefasciatus* | *Culex poicilipes* | *Culex duthoni* | *Aedes hirsutus* | *Aedes aegypti* | *Mansonia africana* | *Mansonia uniformis* | unidentified Culicidae | Total |
| --- | --- | --- | --- | --- | --- | --- | --- | --- | --- | --- | --- | --- | --- | --- | --- | --- | --- | --- | --- | --- | --- | --- | --- | --- | --- |
| RS-2011 | Koubri | 2011 | Indoor | BP  (4) | ♀ | 756 | 728 | 7 | 20 | 0 | 0 | 1 | 426 | 416 | 1 | 0 | 5 | 1 | 0 | 0 | 0 | 2 | 1 | 3 | 1185 |
|  |  |  |  |  | ♂ | 440 | 432 | 0 | 8 | 0 | 0 | 0 | 205 | 203 | 0 | 0 | 2 | 0 | 0 | 0 | 0 | 0 | 0 | 0 | 645 |
|  |  |  |  | SRB-IN (4) | ♀ | 174 | 153 | 5 | 15 | 0 | 0 | 1 | 187 | 155 | 11 | 14 | 0 | 1 | 0 | 0 | 1 | 1 | 4 | 30 | 391 |
|  |  |  |  |  | ♂ | 92 | 84 | 3 | 5 | 0 | 0 | 0 | 46 | 43 | 2 | 1 | 0 | 0 | 0 | 0 | 0 | 0 | 0 | 5 | 143 |
|  |  |  | Outdoor | PIT  (4) | ♀ | 961 | 408 | 453 | 95 | 4 | 1 | 0 | 247 | 201 | 8 | 7 | 10 | 2 | 0 | 1 | 0 | 13 | 5 | 1 | 1209 |
|  |  |  |  |  | ♂ | 1110 | 714 | 339 | 57 | 0 | 0 | 0 | 310 | 297 | 1 | 1 | 11 | 0 | 0 | 0 | 0 | 0 | 0 | 0 | 1420 |
|  |  |  |  | SRB-OUT  (8) | ♀ | 250 | 161 | 7 | 82 | 0 | 0 | 0 | 318 | 269 | 14 | 20 | 0 | 0 | 0 | 0 | 1 | 11 | 3 | 84 | 652 |
|  |  |  |  |  | ♂ | 185 | 132 | 2 | 51 | 0 | 0 | 0 | 168 | 161 | 1 | 5 | 1 | 0 | 0 | 0 | 0 | 0 | 0 | 7 | 360 |
|  | Goden | 2011 | Indoor | BP  (4) | ♀ | 1800 | 1798 | 0 | 2 | 0 | 0 | 0 | 15 | 12 | 3 | 0 | 0 | 0 | 0 | 0 | 0 | 0 | 0 | 0 | 1815 |
|  |  |  |  |  | ♂ | 1125 | 1125 | 0 | 0 | 0 | 0 | 0 | 7 | 7 | 0 | 0 | 0 | 0 | 0 | 0 | 0 | 0 | 0 | 0 | 1132 |
|  |  |  |  | SRB-IN  (4) | ♀ | 275 | 272 | 1 | 2 | 0 | 0 | 0 | 34 | 33 | 1 | 0 | 0 | 0 | 0 | 0 | 0 | 0 | 0 | 13 | 322 |
|  |  |  |  |  | ♂ | 125 | 125 | 0 | 0 | 0 | 0 | 0 | 9 | 9 | 0 | 0 | 0 | 0 | 0 | 0 | 0 | 0 | 0 | 3 | 137 |
|  |  |  | Outdoor | PIT  (4) | ♀ | 1395 | 1284 | 47 | 59 | 2 | 3 | 0 | 101 | 86 | 9 | 3 | 0 | 0 | 0 | 3 | 0 | 0 | 0 | 6 | 1502 |
|  |  |  |  |  | ♂ | 1353 | 1301 | 19 | 33 | 0 | 0 | 0 | 159 | 154 | 2 | 3 | 0 | 0 | 0 | 0 | 0 | 0 | 0 | 21 | 1533 |
|  |  |  |  | SRB-OUT  (8) | ♀ | 618 | 598 | 3 | 17 | 0 | 0 | 0 | 146 | 125 | 16 | 2 | 0 | 0 | 0 | 1 | 2 | 0 | 0 | 54 | 818 |
|  |  |  |  |  | ♂ | 434 | 433 | 0 | 1 | 0 | 0 | 0 | 26 | 25 | 0 | 1 | 0 | 0 | 0 | 0 | 0 | 0 | 0 | 1 | 461 |
| DS-2012 | Goden | 2012 | Indoor | BP  (4) | ♀ | 2240 | 2239 | 0 | 1 | 0 | 0 | 0 | 123 | 107 | 0 | 0 | 0 | 0 | 0 | 16 | 0 | 0 | 0 | 0 | 2363 |
|  |  |  |  |  | ♂ | 509 | 509 | 0 | 0 | 0 | 0 | 0 | 45 | 45 | 0 | 0 | 0 | 0 | 0 | 0 | 0 | 0 | 0 | 0 | 554 |
|  |  |  |  | SRB-IN  (4) | ♀ | 179 | 178 | 0 | 1 | 0 | 0 | 0 | 122 | 118 | 1 | 2 | 1 | 0 | 0 | 0 | 0 | 0 | 0 | 2 | 303 |
|  |  |  |  |  | ♂ | 49 | 49 | 0 | 0 | 0 | 0 | 0 | 58 | 56 | 0 | 2 | 0 | 0 | 0 | 0 | 0 | 0 | 0 | 1 | 108 |
|  |  |  | Outdoor | PIT  (4) | ♀ | 69 | 67 | 1 | 1 | 0 | 0 | 0 | 84 | 68 | 10 | 0 | 0 | 0 | 0 | 6 | 0 | 0 | 0 | 0 | 153 |
|  |  |  |  |  | ♂ | 70 | 69 | 0 | 1 | 0 | 0 | 0 | 68 | 68 | 0 | 0 | 0 | 0 | 0 | 0 | 0 | 0 | 0 | 0 | 138 |
|  |  |  |  | SRB-OUT  (32) | ♀ | 246 | 242 | 0 | 4 | 0 | 0 | 0 | 1334 | 1263 | 12 | 20 | 10 | 0 | 2 | 25 | 2 | 0 | 0 | 50 | 1630 |
|  |  |  |  |  | ♂ | 83 | 83 | 0 | 0 | 0 | 0 | 0 | 587 | 570 | 5 | 7 | 5 | 0 | 0 | 0 | 0 | 0 | 0 | 11 | 681 |
| Total | | | | | | 14538 | 13184 | 887 | 455 | 6 | 4 | 2 | 4825 | 4491 | 97 | 88 | 45 | 4 | 2 | 52 | 6 | 27 | 13 | 292 | 19655 |
