# Supplementary material for: The Sticky Resting Box, a new tool for studying resting behaviour of Afrotropical malaria vectors
Source: Parasit Vectors. 2014 May 29;7:247. doi: 10.1186/1756-3305-7-247 (PMC4049408; doi:10.1186/1756-3305-7-247)
Supplement: Additional file 2 — Daily predicted mean estimates (±SE) of individuals of species of the An. gambiae complex collected by Sticky Resting Box and traditional collection methods. SRB-IN= Sticky resting box indoors; SRB-OUT= Sticky resting box outdoors; BP=Back Pack aspirators indoors; PIT=Pit shelters outdoors; RS-2011=July-December 2011 sampling; DS-2012= April-June 2012 sampling. § = non-significant difference between collection methods. [file 1756-3305-7-247-S2.docx]

## Additional file 2

| Village | Method | Gender | *Anopheles arabiensis* | | | *Anopheles coluzzii* | | | *Anopheles gambiae* s.s. | | |
| --- | --- | --- | --- | --- | --- | --- | --- | --- | --- | --- | --- |
|  |  |  | Median | +SE | -SE | Median | +SE | -SE | Median | +SE | -SE |
| Koubri | BP | ♀ | 0.11 | 0.05 | 0.03 | 2.12 | 0.8 | 0.58 | 0.1 | 0.03 | 0.002 |
| RS-2011 |  | ♂ | 0.05 | 0.03 | 0.02 | 1.26 | 0.63 | 0.42 | 0.05 | 0.02 | 0.01 |
|  | SRB-IN | ♀ | 0.01 | 0.01 | 0.007 | 0.35 | 0.14 | 0.1 | 0 | - | - |
|  |  | ♂ | 0.002 | 0.006 | 0.002 | 0.2 | 0.1 | 0.06 | 0.005 | 0.009 | 0.003 |
|  | PIT | ♀ | 0.37 | 0.21 | 0.13 | 1.73 | 0.65 | 0.47 | 0.14 | 0.11 | 0.06 |
|  |  | ♂ | 0.42 | 0.29 | 0.17 | 2.08 | 1.22 | 0.77 | 0.29 | 0.18 | 0.11 |
|  | SRB-OUT | ♀ | 0.03 | 0.02 | 0.01 | 0.3 | 0.11 | 0.08 | 0.006 | 0.008 | 0.003 |
|  |  | ♂ | 0.007 | 0.008 | 0.004 | 0.13 | 0.08 | 0.05 | 0 | - | - |
| Goden | BP | ♀ | 0.38 | 0.14 | 0.1 | 2.89 | 0.76 | 0.6 | 0.09 | 0.02 | 0.02 |
| RS-2011 |  | ♂ | 0.11 | 0.07 | 0.04 | 1.56 | 0.8 | 0.53 | 0.02 | 0.01 | 0.008 |
|  | SRB-IN | ♀ | 0.12 | 0.05 | 0.03 | 0.85 | 0.23 | 0.18 | 0.05^§^ | 0.02 | 0.01 |
|  |  | ♂ | 0.003 | 0.005 | 0.002 | 0.26 | 0.14 | 0.09 | 0.02^§^ | 0.01 | 0.01 |
|  | PIT | ♀ | 2.2 | 0.68 | 0.52 | 2.98 | 0.69 | 0.56 | 0.31 | 0.1 | 0.07 |
|  |  | ♂ | 1.43 | 0.58 | 0.41 | 2.99 | 0.83 | 0.65 | 0.15^§^ | 0.06 | 0.04 |
|  | SRB-OUT | ♀ | 0.36 | 0.11 | 0.09 | 1.85 | 0.42 | 0.34 | 0.09 | 0.03 | 0.02 |
|  |  | ♂ | 0.12 | 0.05 | 0.03 | 1.27 | 0.35 | 0.27 | 0.11 | 0.04 | 0.03 |
| Goden | BP | ♀ | 0.95 | 0.33 | 0.24 | 3.98 | 1.78 | 1.23 | 0 | - | - |
| DS-2012 |  | ♂ | 0.17 | 0.05 | 0.03 | 0.66 | 0.37 | 0.24 | 0 | - | - |
|  | SRB-IN | ♀ | 0.76 | 0.03 | 0.02 | 0.59 | 0.27 | 0.18 | 0 | - | - |
|  |  | ♂ | 0.009 | 0.01 | 0.005 | 0.12 | 0.07 | 0.04 | 0 | - | - |
|  | PIT | ♀ | 0.2 | 0.09 | 0.06 | 0.22 | 0.11 | 0.07 | 0 | - | - |
|  |  | ♂ | 0.15 | 0.09 | 0.05 | 0.15 | 0.1 | 0.06 | 0 | - | - |
|  | SRB-OUT | ♀ | 0.06 | 0.02 | 0.01 | 0.14 | 0.06 | 0.04 | 0 | - | - |
|  |  | ♂ | 0.01 | 0.01 | 0.007 | 0.03 | 0.02 | 0.01 | 0 | - | - |
