# Supplementary material for: The Sticky Resting Box, a new tool for studying resting behaviour of Afrotropical malaria vectors
Source: Parasit Vectors. 2014 May 29;7:247. doi: 10.1186/1756-3305-7-247 (PMC4049408; doi:10.1186/1756-3305-7-247)
Supplement: Additional file 3 — Performance of SRB adhesive sheets over time. Percentage of An. coluzzii females stuck on sticky resting boxes exposed to outdoor conditions for 1, 3, 5 and 7 days. Chi-square and p-values for pair comparisons are reported. [file 1756-3305-7-247-S3.docx]

**Additional file 3**

| Days | Cage 1 | Cage 2 | Cage 3 | N |
| --- | --- | --- | --- | --- |
| 1 | 49% | 35% | 68% | 300 |
| 3 | 61% | 40% | 66% | 300 |
| 5 | 40% | 56% | 54% | 300 |
| 7 | 65% | 60% | 67% | 300 |
| Total | 54% | 48% | 64% | 1200 |
| Total χ^2^ = 15.18 (3 DF); P= 0.002 | | | | |
| D1 vs D3 | χ2 = 2.91; P = 0.09 | χ2 = 0.53; P = 0.47 | χ2 = 0.09; P = 0.76 |  |
| D1 vs D5 | χ2 = 1.64; P = 0.20 | χ2 = 8.89; P = 0.003 | χ2 = 4.12; P = 0.04 |  |
| D1 vs D7 | χ2 = 5.22; P = 0.02 | χ2 = 12.5; P < 0.001 | χ2 = 0.02; P = 0.88 |  |
